# 2095 Drug screening and hit identification for night blindness with zebrafish

**DOI:** 10.1017/cts.2018.69

**Published:** 2018-11-21

**Authors:** Logan Ganzen, Yuk Fai Leung

## Abstract

OBJECTIVES/SPECIFIC AIMS: Retinitis pigmentosa (RP), also known as night blindness, is an incurable disease which affects ~1 in 4000 individuals globally. Since there are no effective treatment options for RP, the goal of this project is to identify novel drug treatments that can prevent or slow the disease progression. To this end, we optimized a behavioral assay, visual-motor-response (VMR) assay, to investigate rod function (Ganzen *et al*., *ARVO*, 2017; Ganzen *et al*., *IJMS*, 2017). This was done utilizing a transgenic zebrafish RP model expressing human rhodopsin with the Q344X mutation. In this study, we used this model to perform a proof-of-concept screen for drugs which may improve the vision of the larvae. METHODS/STUDY POPULATION: To screen for beneficial drugs, the SCREEN-WELL^®^ REDOX library was chosen for screening. This library was selected to identify a compound that may alleviate any excessive oxidative stress in the diseased retina. The Q344X zebrafish line suffers from significant rod degeneration by 7 days postfertilization (dpf) and displayed deficits in VMR under scotopic conditions (Ganzen *et al.*, *ARVO*, 2017). The Q344X larvae were drug treated beginning at 5 dpf at 10 μM. Compounds that were toxic at this concentration were retested at 1 μM. The 5 dpf stage was chosen as most of the rods are intact, and these concentrations were chosen to optimize the drug effect based on similar studies. Hits were identified by assays that provided a robust and reproducible enhancement in the Q344X VMR. The retinae of any drug hits were dissected from larvae crossed with a rod EGFP reporter line and whole-mounted to analyze rod survival via fluorescence. To determine if drug effects were exerted through the retina, eyeless chokh mutant zebrafish were exposed to the drug and tested with the same assay. RESULTS/ANTICIPATED RESULTS: Of the 84 compounds tested, we identified 1 drug that ameliorated the VMR of the Q344X scotopic VMR. Eyeless chokh mutant zebrafish larvae did not exhibit the same VMR when treated with the same drug. Histological analysis suggested increased rod survival in the drug-treated retina of Q344X mutants. DISCUSSION/SIGNIFICANCE OF IMPACT: These results indicate that the vision of the Q344X zebrafish was improved via this beneficial drug treatment. Since eyeless chokh larvae did not respond to the same treatment, the drug likely mediated its positive effects through the Q344X retina, likely by improving rod survival. Together, our results have identified a beneficial drug that may treat RP.

